# Sickness absence among privately employed white-collar workers: A total population study in Sweden

**DOI:** 10.1177/1403494820934275

**Published:** 2020-07-10

**Authors:** Kristin Farrants, Kristina Alexanderson

**Affiliations:** Division of Insurance Medicine, Department of Clinical Neuroscience, Karolinska Institutet, Sweden

**Keywords:** Sick leave, sickness absence, disability pension, white-collar workers, private sector

## Abstract

*Background*: Knowledge about sickness absence (SA) and disability pension (DP) among privately employed white-collar workers is very limited. *Aims*: This study aimed to explore SA and DP among privately employed white-collar women and men using different measures of SA to investigate differences by branch of industry, and to analyse the association between sociodemographic factors and SA. *Methods*: This was a population-based study of all 1,283,516 (47% women) privately employed white-collar workers in Sweden in 2012, using register data linked at the individual level. Several different measures of SA and DP were used. Logistic regression was used to investigate associations of sociodemographic factors with SA. *Results*: More women than men had SA (10.9% women vs. 4.5% men) and DP (1.8% women vs. 0.6% men). While women had a higher risk of SA than men and had more SA days per employed person, they did not have more SA days per person with SA than men. The risk of SA was higher for women (odds ratio (OR)=2.54 (95% confidence interval (CI) 2.51–2.58)), older individuals (OR age 18–24 years=0.58 (95% CI 0.56–0.60); age 55–64 years OR=1.43 (95% CI 1.40–1.46) compared to age 45–54 years), living in medium-sized towns (OR=1.05 (95% CI 1.03–1.06)) or small towns/rural areas (OR=1.13 (95% CI 1.11–1.15)), with shorter education than college/university (OR compulsory only=1.64 (95% CI 1.59–1.69); OR high school=1.38 (95% CI 1.36–1.40)), born outside the EU25 (OR=1.23 (95% CI 1.20–1.27)) and singles with children at home (OR=1.33 (95% CI 1.30–1.36)). ***Conclusions*: SA and DP among privately employed white-collar workers were lower than in the general population. SA prevalence, length and risk varied by branch of industry, sex and other sociodemographic factors, however, depending on the SA measure used**.

## Introduction

In recent years, the number of studies on sickness absence (SA) in general and in specific occupations and diagnoses has increased. However, knowledge about SA among privately employed white-collar workers is still very limited, despite this constituting a very large group in most countries. In Sweden, approximately half the workforce were white-collar workers in 2018 [1].

The two largest study cohorts of white-collar employees are The Whitehall-II study of British civil servants, of whom the majority are white-collar workers [2,3], and the Helsinki Health Study of municipal workers, including both white-collar and blue-collar workers [4]. These studies of public employees show that there are SA differences within the white-collar workers by age, sex, education, occupational status and other sociodemographic and socio-economic factors [3,5].

Regarding privately employed white-collar workers, knowledge is substantially more limited. Some reports in Swedish regarding SA among white-collar workers have been published. However, only a few results were stratified for public and private employees [6–8]. Furthermore, all those studies are based on surveys with response rates of 21–49% [6–9]. Thus, a nationwide study of all privately employed white-collar workers in Sweden based on high-quality register data can add to the knowledge base of the situation in this under-studied group.

White-collar workers generally have lower SA rates than blue-collar workers [5,10]. While most previous research has focused on groups with high SA rates, it is also important to gain knowledge about groups with lower rates, as they constitute large parts of the labour market, meaning that their SA has great implications for their companies, society and themselves.

The Swedish Social Insurance Agency found differences in SA prevalence between branches of industry that cannot be fully explained by differences in occupational structures [11]. Whether these differences can also be found among white-collar workers is not known.

In most countries, women have higher SA rates than men [12]. Possible explanations for this include: (a) higher morbidity rates among women, especially the types of morbidity leading to SA [13,14]; (b) scientific knowledge regarding diagnosis, treatment, prevention and rehabilitation measures for women being more limited; and (c) women having more ergonomically or psychosocially demanding paid and unpaid work [15].

There are currently more than 100 different measures of SA in the literature [16]. These mirror the challenges of SA, such as recurring events of different durations and grade, skewed distributions, that both incidence and duration matters and so on. Measures are based on both different numerators (spells, days, individuals, etc.) and different denominators (individuals at work, insured individuals, total individuals in the population, etc.). Different measures will lead to different results in the same data (e.g. regarding sex differences in SA) [17].

The aims were to explore SA and DP among privately employed white-collar women and men using different measures of SA, to investigate differences by branch of industry and to analyse the association between sociodemographic factors and SA.

## Methods

This was a population-based study of SA and DP among privately employed white-collar workers undertaken during 2012 using different SA measures.

### Data and study population

We used data from two nationwide Swedish administrative registers linked at the individual level by use of the personal identity number (PIN; a unique 10-digit number assigned to all Swedish residents): (a) the Longitudinal Integration Database for Health Insurance and Labour Market Studies (LISA) held by Statistics Sweden to identify the source population and for information on age, sex, country of birth, type of living area, family situation, educational level, income, occupational code, sector and branch of industry; and (b) the MicroData for Analysis of the Social Insurance database (MiDAS) held by the Social Insurance Agency for information on SA spells >14 days (dates, extent, main diagnosis) and DP (dates and extent).

The study population comprised all those who were aged 18-67 years and registered as living in Sweden on 31 December 2011 and 31 December 2012, who had an occupational code according to the Swedish Standard for Occupational Classification that indicated a white-collar occupation, were employed at a private-sector company and during 2012 had income from work, parental benefits and/or SA/DP that amounted to at least 7920 SEK (i.e. 75% of the necessary income level to qualify for SA benefits from the Social Insurance Agency). The limit of 75% of the minimum income to qualify for SA benefits was set, since in many cases, SA benefit is about 75% of the work income; without this adjustment, people with low incomes and long-term SA might have fallen below the minimum income level to be included in the study [18,19]. Those who were employed in the public sector, self-employed or who had full-time DP for the whole of 2012 were excluded. The total study population was 1,283,516 individuals.

### Variables

We used information on the following variables: sex (woman or man); age (18–24, 25–34, 35–44, 45–54, 55–64, or 65–67 years); country of birth (Sweden, other Nordic country, other EU25 or rest of world, including missing values); educational level (compulsory school (⩽9 years or missing), high school (10–12 years) or college/university (⩾13 years)); family situation (married/cohabiting with children at home, married cohabiting without children at home, single with children at home or single without children at home); type of living area (large city (Stockholm, Gothenburg or Malmö), medium-sized town (>90,000 inhabitants within 30 km of city centre) or small town/rural (<90,000 inhabitants within 30 km of city centre or rural)); branch of industry based on the Swedish Standard for Industry Classification (SNI) categorised into the following six groups: manufacturing, services, transport, construction and installation, care and education, or commerce and hospitality. There were 1567 individuals for whom information on branch of industry was missing. For these individuals, we used information on occupation to classify the branch of industry where possible, with 1001 individuals having their branch of industry assigned this way. The remaining 566 individuals, for whom it was not possible to assign a branch of industry, are presented in Table I but not included in any further analyses.

**Table I.** Sociodemographic characteristics of the privately employed white-collar workers in Sweden in 2012, both in total and stratified by sex.

Sociodemographic characteristics	Total		Women		Men	
	*n*	%	*n*	%	*n*	%
All	1,283,516	100	608,793	100	674,723	100
*Sex*						
Women	608,793	47.4				
Men	674,723	52.6				
*Type of living area*						
Large city	662,644	51.6	325,832	53.5	336,812	49.9
Medium-sized town	391,236	30.5	176,002	28.9	215,234	31.9
Small town/rural	229,636	17.9	106,959	17.6	122,677	18.2
*Age (years)*						
18–24	65,090	5.1	37,598	6.2	27,492	4.1
25–34	277,169	21.6	138,712	22.8	138,457	20.5
35–44	377,221	29.4	176,930	29.1	200,291	29.7
45–54	327,936	25.5	152,163	25.0	175,773	26.1
55–64	216,007	16.8	95,641	15.7	120,366	17.8
65–67	20,093	1.6	7749	1.3	12,344	1.8
*Education (years)*						
Compulsory (0–9)	63,149	4.9	23,860	3.9	39,289	5.8
Secondary (10–12)	529,675	41.3	266,672	43.8	263,003	39.0
College/university (>12)	690,692	53.8	318,261	52.3	372,431	55.2
*Country of birth*						
Sweden	1,148,760	89.5	536,421	88.1	612,339	90.8
Other Nordic	27,938	2.2	16,229	2.7	11,709	1.7
Other EU25	26,437	2.1	13,255	2.2	13,182	2.0
Rest of world	80,381	6.3	42,888	7.0	37,493	5.6
*Family situation*						
Married/cohabiting without children at home	172,351	13.4	79,654	13.1	92,697	13.7
Married/cohabiting with children at home	603,434	47.0	276,042	45.3	327,392	48.5
Single without children at home	422,531	32.9	190,960	31.4	231,571	34.3
Single with children at home	85,200	6.6	62137	10.2	23,063	3.4
*Branch of industry*						
Manufacturing	265,252	20.7	85,547	14.1	179,705	26.6
Services	554,399	43.2	254,898	41.9	299,501	44.4
Commerce and hospitality	164,224	12.8	85,984	14.1	78,240	11.6
Transports	56,087	4.4	20,979	3.4	35,108	5.2
Construction and installation	50,671	3.9	14,357	2.4	36,314	5.4
Care and education	192,319	15.0	146,851	24.1	45,466	6.7
Missing	566	<0.1	177	<0.1	389	<0.1

### Measures

We calculated the following different measures of SA and DP in this study:

- numbers and one-year period prevalence (hereafter; prevalence) of people with SA;
- numbers and prevalence of people with DP;
- mean number of net days with SA and DP per person;
- mean number of gross days of SA per person;
- mean number of gross days of SA per person with SA;
- mean number of net days of SA per person;
- mean number of net days of SA per person with SA;
- median number of gross days with SA per person with SA; and
- median number of net days with SA per person with SA.

For all the above 11 measures, the quotient between women and men was also calculated. We also calculated:

- odds ratios of the risk of SA in a subgroup, relative to the risk in a reference group.

For the calculation of net days, part-time SA was combined (e.g. two days of 50% SA or DP were combined to one net day) in order to handle the possibility of part-time SA/DP.

We conducted statistical descriptive and epidemiological analyses, and present the results for all as well as stratified by sex. We also conducted logistic regression to determine the difference in risk for SA in by the above variables, controlling for other factors.

The project was approved by the Regional Ethical Review Board of Stockholm, Sweden.

### Public SA insurance in Sweden

All people living in Sweden aged ⩾16 years with an income from work or unemployment benefit who due to disease or injury have a reduced work capacity are covered by the national public SA insurance, providing SA benefits. After a first qualifying day, the employer pays sick pay for the first 14 days of a SA spell. Thereafter, SA benefits are paid by the Social Insurance Agency. The self-employed have more qualifying days. The unemployed get SA benefits from the Social Insurance Agency after the first qualifying day. A physician’s certificate is required after seven days of self-certification. In this study, data on SA with benefits from the Social Insurance Agency were used. SA spells <15 days were not included in the study, so as not to introduce bias regarding those who might have been unemployed part of the year of 2012, and net days in SA spells ⩾15 days were only counted from day 15. All residents in Sweden aged 19–64 years whose work capacity is permanently or reduced long term due to disease or injury can be granted DP from the Social Insurance Agency.

Both SA and DP can be granted for part- or full-time (25%, 50%, 75% or 100% of ordinary work hours). SA benefits cover 80% and DP benefits 64% of lost income, both up to a certain level.

## Results

Table I shows the sociodemographic make-up of the study population of 1,283,516 privately employed white-collar workers. Roughly half the population (53.5% of the women and 49.9% of the men) lived in a large city. The 35–44 age category was the largest for both women and men. The absolute majority had more than compulsory education (95.1% of the women and 94.2% of the men), and more than half had at least some college/university education (52.3% of the women and 55.2% of the men). The largest branch of industry in this population was services (41.9% of the women and 44.4% of the men), and the smallest was construction and installation (3.5% of the women and 5.4% of the men). Information about branch of industry was missing for 566 individuals (<0.1% of the study population).

Table II shows numbers and prevalence rates of individuals with SA and DP, respectively, during 2012. Almost 11% of the women had at least one SA spell >14 days, which is more than twice as high as among men (4.5%). Similarly there was a higher prevalence of DP to some extent during 2012 among women (1.8%) than men (0.6%).

**Table II.** Numbers and one-year prevalence (%) of people with a sickness absence spell lasting >14 days and of people with disability pension during 2012.

	Sickness absence		Disability pension	
	*n*	%	*n*	%
Total	97,102	7.6	14,911	1.2
Women	66,410	10.9	10,762	1.8
Men	30,692	4.5	4149	0.6

Table III shows several measures of SA and DP for women and men, as well as the quotient between women’s and men’s values. The quotient for the prevalence of SA between women and men was 2.42, and the quotient for the prevalence of DP was 3.00, meaning that women had more than twice the risk of SA and three times the risk of DP. Women also had more than twice as many gross and net SA days per employed than men did (quotient of 2.45 for gross days and 2.37 for net days). However, for the mean number of days with SA per person with SA, the quotient between women and men was very close to 1.

**Table III.** Compilation of 10 different measures of sickness absence (SA) and disability pension (DP) for among privately employed white-collar workers in Sweden (*N*=1,283,516), among all, stratified by women and men, and quotient of values between women and men.

	One-year period prevalence of SA (%)	Mean no. gross days SA/employed	Mean no. net days SA/employed	Mean no. gross days SA/person with SA	Mean no. net days SA/person with SA	One year period prevalence of DP (%)	Mean no. gross days SA+DP/employed	Mean no. net days SA+DP/employed	Median no. gross days SA/person with SA	Median no. net days SA/person with SA
Total	7.60	6.08	4.60	80.16	60.68	1.20	10.09	6.38	40	31.00
Women	10.90	8.81	6.60	80.76	60.48	1.80	14.95	9.31	41	31.00
Men	4.50	3.59	2.78	78.86	61.11	0.60	5.69	3.74	38	30.50
Quotient women/men	2.42	2.45	2.37	1.02	0.99	3.00	2.63	2.49	1.08	1.02

**Figure 1.**
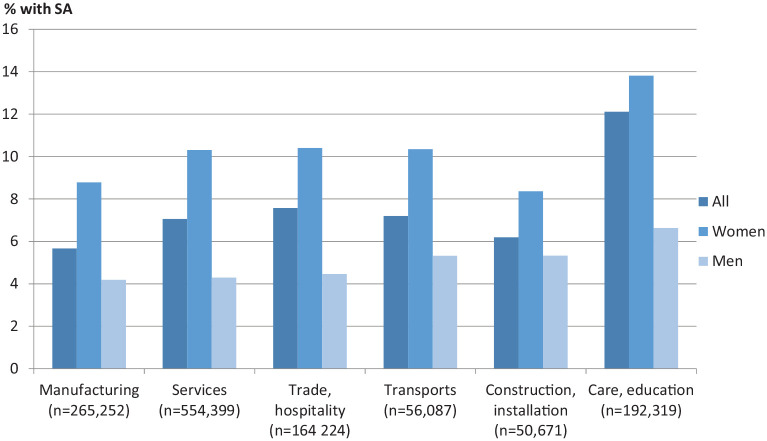
One-year period prevalence (%) of any sickness absence (SA) spell >14 days in 2012 among privately employed white-collar workers (*N*=1,283,516) by branch of industry.

Figure 1 shows the percentage of individuals with SA during 2012 in each branch of industry. In all six branches of industry, men had a lower prevalence of SA than women. Care and education had the highest prevalence of SA among both women (13.8%) and men (6.6%). For women, construction and installation had the lowest prevalence (8.4%), while for men, manufacturing had the lowest (5.7%).

We also performed logistic regression analyses for the odds ratios (OR) of SA between groups, controlling for the six sociodemographic variables (Table IV). We found a statistically significant association between the risk of SA and all included variables in the analysis when adjusting for the other variables. Women had a significantly and substantially higher risk of SA than men (OR=2.54). The risk for SA was also higher in the older age groups than in the younger age groups, with the exception of the very oldest (65–67 years). Those who were aged <25 years and >64 years had ORs <1 compared to those aged 35–44 (OR=0.58 and 0.72, respectively). Those with lower education had a significantly and substantially higher OR of SA than those with at least some college/university education. Those born in other Nordic countries and outside the EU25 had somewhat higher ORs of SA than those born in Sweden, but there was no significant difference between those born in Sweden and those born in the EU25 excluding the Nordic countries.

**Table IV.** Crude and adjusted odds ratios (OR) and 95% confidence interval (CI) over the risk of having at least one SA spell >14 days by sex, type of living area, age, education, country of birth and family situation among privately employed white-collar workers in Sweden (*N*=1,283,516).

	Total				Women				Men			
	Crude OR	95% CI	Adjusted OR	95% CI	Crude OR	95% CI	Adjusted OR	95% CI	Crude OR	95% CI	Adjusted OR	95% CI
*Sex*												
Women	2.57	(2.53–2.61)	2.54	(2.51–2.58)								
Men	Ref.		Ref.									
*Type of living area*												
Large city	0.66	(0.64–0.69)	0.58	(0.56–0.60)	0.53	(0.51–9.57)	0.58	(0.56–0.61)	0.82	(0.76–0.88)	0.59	(0.55–0.64)
Medium-sized town	0.99	(0.70–1.01)	1.02	(1.00–1.04)	1.01	(0.98–1.03)	1.13	(1.10–1.16)	0.81	(0.78–0.84)	0.75	(0.72–0.78)
Small town	Ref.		Ref.		Ref.		Ref.		Ref.		Ref.	
*Age (years)*												
18–24	1.10	(1.08–1.12)	1.05	(1.04–1.07)	1.00	(0.98–1.02)	0.97	(0.95–1.00)	1.35	(1.30–1.39)	1.28	(1.23–1.32)
25–34	1.42	(1.39–1.45)	1.43	(1.40–1.46)	1.20	(1.17–1.23)	1.27	(1.23–1.30)	2.07	(2.00–2.13)	1.80	(1.74–1.87)
35–44	0.67	(0.63–0.72)	0.72	(0.67–0.76)	0.50	(0.46–0.55)	0.56	(0.51–0.61)	1.16	(1.06–1.27)	0.98	(0.89–1.07)
45–54	Ref.		Ref.		Ref.		Ref.		Ref.		Ref.	
55–64	1.03	(1.02–1.05)	1.05	(1.03–1.06)	1.04	(1.02–1.06)	1.03	(1.01–1.05)	1.14	(1.11–1.17)	1.07	(1.04–1.10)
65–67	1.17	(1.15–1.19)	1.13	(1.11–1.15)	1.13	(1.11–1.16)	1.10	(1.07–1.12)	1.35	(1.13–1.39)	1.17	(1.14–1.21)
*Education (years)*												
Compulsory (0–9)	1.62	(1.56–1.67)	1.64	(1.59–1.69)	1.49	(1.44–1.55)	1.48	(1.43–1.54)	2.23	(2.14–2.32)	1.86	(1.78–1.93)
Secondary (10–12)	1.45	(1.43–1.47)	1.38	(1.36–1.40)	1.31	(1.29–1.33)	1.33	(1.31–1.36)	1.59	(1.56–1.63)	1.50	(1.46–1.54)
College/university (>12)	Ref.		Ref.		Ref.		Ref.		Ref.		Ref.	
*Country of birth*												
Sweden	Ref.		Ref.		Ref.		Ref.		Ref.		Ref.	
Other Nordic	1.31	(1.26–1.37)	1.09	(1.05–1.14)	1.14	(1.09–1.20)	1.09	(1.04–1.15)	1.34	(1.24–1.45)	1.16	(1.08–1.26)
Other EU25	1.02	(0.98–1.07)	1.03	(1.05–1.08)	0.99	(0.94–1.05)	1.01	(0.96–1.07)	1.00	(0.92–1.09)	1.10	(1.01–1.19)
Rest of world	1.22	(1.19–1.25)	1.23	(1.20–1.27)	1.16	(1.13–1.20)	1.18	(1.15–1.22)	1.14	(1.08–1.19)	1.35	(1.29–1.42)
*Family situation*												
Married/cohabiting without children at home	1.17	(1.15–1.20)	0.94	(0.92–0.96)	0.97	(0.95–1.00)	0.84	(0.82–0.87)	1.64	(1.58–1.69)	1.17	(1.12–1.21)
Married/cohabiting with children at home	Ref.		Ref.		Ref.		Ref.		Ref.		Ref.	
Single without children at home	0.93	(0.91–0.94)	0.96	(0.95–0.98)	0.81	(0.79–0.83)	0.83	(0.81–0.85)	1.21	(1.17–1.24)	1.34	(1.30–1.38)
Single with children at home	1.77	(1.73–1.81)	1.33	(1.30–1.36)	1.33	(1.30–1.37)	1.29	(1.25–1.32)	1.56	(1.41–1.65)	1.34	(1.27–1.42)

Adjusted OR are mutually adjusted for all other factors in the analysis.

Patterns for women and men were generally similar, with some minor differences. The age differences were larger among men than among women. Women aged 65–67 years had a lower risk of SA than those aged 35–44 years (OR=0.56), while the difference between these two age categories was insignificant for men. Men born outside Sweden had higher risks for SA, but for women, there was no significant difference between those born in Sweden and those born in the EU25, excluding the Nordic countries.

## Discussion

In this exploratory study of SA among 1.3 million privately employed white-collar workers in Sweden in 2012, we found that the risk of SA was higher for women, older individuals (except those aged 65–67 years), those living in small towns or rural areas, individuals with shorter education, those born outside the EU25 and singles with children at home.

SA is a very complex phenomenon that can be measured in different ways [16]. That is why we used several different SA measures in this study, related both to prevalence, length and OR of SA. These measures showed different results. For instance, the sex differences regarding prevalence of SA and DP, as well as regarding SA days per employed person, were marked. The prevalence rates of SA and DP, as well as mean number of days with SA and/or DP, were twice as high for women than for men. However, the mean and median numbers of days of SA per person with SA were very similar between women and men. This indicates that while women had a higher risk of SA than men, they did not have more SA days per person on SA.

There were differences in SA by branch of industry among both women and men. Care and education had the highest SA prevalence for both women and men. A previous study also found that there is a higher risk of SA in these branches of industry, as well as in others with close contact with customers, clients and patients [20].

Since the Swedish labour market is highly gender segregated [21], some have argued that part of the reason why women have more SA than men is that there are more women in occupations with high SA prevalence [22]. However, we found that women’s SA prevalence was higher in all six branches of industry. Some studies have found that being the minority sex in a highly sex-segregated occupation is associated with higher risk of SA [23]. For both women and men, the branch of industry with the highest SA prevalence was care and education, and women did not have higher SA prevalence in manufacturing or construction, comprising many numerically male-dominated occupations. However, as we specifically investigated white-collar workers, it is likely that women in manufacturing and construction are involved in either management or clerical work, and clerical work in particular is not a male-dominated occupation.

Those living in large cities (Stockholm, Gothenburg or Malmö) had a lower risk of SA than those in medium-sized or small towns. This is in line with previous research, and might reflect that it is easier to find an alternative job if it is not possible to remain in the current job due to reduced work capacity [24], or a possible higher rate of older people with worse somatic and mental health in more rural areas [25]. We also found that those born in Sweden had a lower risk of SA, which is in line with previous results [26].

We also found that women without children living at home had a lower risk of SA, while this was not the case for men. A study of white-collar women in Sweden found that those who were on SA reported more difficulties in combining work and family life [27].

The Swedish Social Insurance Agency measures SA and DP by net days per insured person. In our study, privately employed white-collar workers had 4.6 net days per employed person (6.6 for women and 2.8 for men; Table III). According to statistics from the Swedish Social Insurance Agency, the corresponding number among all employed and self-employed in Sweden was 6.9 net days per person in 2012, 9.0 for women and 4.8 for men [28]. Privately employed white-collar workers thus also had fewer net days per person than the national average for employed and self-employed persons. That white-collar employees have lower SA than the general population is not surprising. White-collar workers have lower risk of morbidity [29], and they are more able to work with certain health conditions, for example, as their work is seldom physically demanding [29].

### Strengths and limitations

The main strength of this study is its large cohort: it included all 1.3 million individuals who lived in Sweden throughout 2012, who were 18–67 years old and who were employed in a white-collar occupation by a private company. This means that the study is not based on a sample, and also that the study population was large enough for subgroup analysis. Another important strength is that good-quality data from two nationwide registers [30] linked at the individual level were used. Several analyses have been conducted, and several measures of both numbers and prevalence of individuals as well as of days were used to describe and analyse SA and DP in this population as a basis for further studies.

Limitations are the cross-sectional and exploratory nature of the study, meaning that we were unable to draw any causal inferences from the research. That we only used SA spells >14 days can be seen as both a strength and a limitation.

## Conclusion

There has to date been very little research on SA in privately employed white-collar employees. In this first explorative study, we have shown that while women have a higher risk of SA than men, there are no sex differences in length of SA among those on SA. We have also shown that there are differences in SA between privately employed white-collar employees related to both their sociodemographic factors and to their branch of industry. The magnitude of sex differences in SA varied with SA measure used, indicating the need for several measures to portray the complexity of the phenomenon fully.
